# Experience and Perceptions of Chinese University Students Regarding the COVID-19 Pandemic: A Qualitative Analysis

**DOI:** 10.3389/fpubh.2022.872847

**Published:** 2022-05-03

**Authors:** Yijin Wu, Gaohui Yin, Yichi Zhang

**Affiliations:** Center for Medical Humanities in the Developing World, School of Translation Studies, Qufu Normal Univeristy, Rizhao, China

**Keywords:** COVID-19, experiences, perceptions, university students, China, content analysis

## Abstract

The COVID-19 pandemic has exerted a significant influence on university student's learning, life and mental health. Using the method of inductive content analysis, this study examined the experience and perceptions of Chinese university students regarding the COVID-19 pandemic. Eighteen university students including seven males and eleven females were involved in this study. In this study, we identified five themes concerning experience and perceptions of Chinese university students regarding the COVID-19 pandemic, that is, emotional reactions toward the COVID-19 pandemic, the influence of the COVID-19 pandemic on the participant's learning, the influence of the COVID-19 pandemic on the participant's daily life, participants' positive responses to the COVID-19 pandemic, and China's moves toward the COVID-19 pandemic. This study elaborated on experience and perceptions of Chinese university students regarding the COVID-19 pandemic, which would provide us a better understanding of how university students perceive the COVID-19 pandemic and to what extent the COVID-19 pandemic has influenced their learning and daily lives.

## Introduction

Since December 2019, the 2019 novel Coronavirus (2019-nCoV) has been spreading rapidly across cities in China and around the world (1, 2). More than 4.86 million confirmed cases were reported and 6.14 million death cases emerged among over 200 counties and regions (3). With the rapidly growing number of infected people across the globe, the pandemic was declared as a public health emergency by the World Health Organization (WHO) on 30 January 2020 (4). As of April 13, 2020, the official website of the National Health Commission of China reported that there had been 82,249 confirmed cases and 1,464 imported cases of the COVID-19 pandemic in China (4). The COVID-19 pandemic not only had enormous impact on China's economy, but also had devastating impact on all levels of education (5, 6). All universities in China were in lockdown during the first outbreak of the COVID-19. According to the latest figures released by Ministry of Education of the People's Republic of China, 50 million university students in China were not able to attend university (7). University education changed dramatically and teaching moved online. It has been suggested that online learning is effective only if students have consistent access to the Internet and computers for online learning and if teachers have received special online instruction training (8). University students could have easy access to the Internet and computers and teachers could have a good command of online instruction in developed countries (9). However, students in developing countries may lack consistent access to the Internet or computers and teachers could not receive adequate training to teach online. In addition, the pandemic had a negative influence on university students' mental health (5, 10). It has been found that university students seem to be more likely to be influenced by mental illnesses during COVID-19 pandemic in France (11). Specifically, they are susceptible to anxiety, depression and post-traumatic stress (12).

The influence of the COVID-19 pandemic on the university student's learning, life and mental health have been extensively examined in developed countries (13, 14). Little is known about how and to what extent the COVID-19 pandemic influence the university student's learning, daily life and mental health and what types of perceptions they hold toward COVID-19 pandemic in developing countries, especially in China. In this study, using the method of thematic content analysis, we examined the experience and perceptions of Chinese university students toward the COVID-19 pandemic. To the best of our knowledge, this is the first qualitative study to explore the experience and perceptions of Chinese university students regarding the COVID-19 pandemic.

## Methods

### Study Design

The study design was a qualitative investigation involving semi-structured interviews (15–17). From April 10 to April 24, 2020, telephone interviews were conducted to investigate the experiences and perceptions of Chinese university students toward the COVID-19 pandemic (18). This study provided detailed descriptions of experience and perceptions of Chinese university students regarding the COVID-19 pandemic by comparing the similarities and differences between coded data (19–22). An inductive approach was chosen for presenting original, first-hand, qualitative information on experience and perceptions of Chinese university students regarding the COVID-19 pandemic (23). This study aimed to answer the following two questions: What are the experience and perceptions of Chinese university students concerning the COVID-19 pandemic in China? How does the COVID-19 pandemic influence the quality of life and learning outcomes among Chinese university students? The results of this study were presented in accordance with the consolidated criteria for reporting qualitative research (CO-REQ) (24).

### Study Participants

Eighteen university students including seven males and 11 females were involved in this study. In order to compensate for the small size of the sample, we purposefully recruited participants from different cities with ages from 18 to 23 years old. The mean age of participants was 21.5 years. All participants had the ability to speak and write Chinese and were able to complete semi-structured interviews and describe their experience and perceptions toward the COVID-19 pandemic. Informed consent was obtained from all participants prior to data collection.

### Data Collection

Purposive sampling method was used to recruit participants. Participants involved in this study were university students who experienced the COVID-19 pandemic. Data collection stopped at a point where no new themes emerged. A total number of 18 participants were interviewed based on the interview guide (Table 1). Participants were encouraged to talk about their experience and perceptions of the COVID-19 pandemic. Interview questions began with the participant's emotional experiences, then expanded to their experience and perceptions of online learning and daily lives, and finally focused on their reactions to the COVID-19 pandemic. A pilot interview was conducted to ensure that the collected data are relevant to the research questions. All the interviews were audio-recorded and lasted 13–40 mins, which relied on how much experience they had about the COVID-19 pandemic and what kind of perception they would have about the COVID-19 pandemic. The mean length of interviews was 20 min. All semi-structured interviews were conducted via phone in order to prevent cross-infection between interviewers and interviewees.

**Table 1:** Interview guide.

1	How was your psychological process during the COVID-19 pandemic?
2	How did the COVID-19 pandemic influence your learning?
3	How did the COVID-19 pandemic influence your life?
4	What did you do in response to the COVID-19 pandemic?
5	How does China fight against the COVID-19 pandemic?

### Data Analysis

The method of Inductive Content Analysis was used to analyze the collected data (25). This study takes up the following analytical procedures. First, we performed a verbatim transcription of interview data. Second, all the transcriptions were verified independently by all the researchers to ensure the accuracy of data transcription. Third, all researchers discussed open coding fully after identifying meaningful words, phrases, and sentences in the transcribed data. Open coding was performed through each transcript by all researchers to create categories, rather than simply summarizing the interviewees' opinions (26). Meanings were conveyed in terms of themes and subthemes in this study (27). Third, the leading investigator summarized the codings concerning the experience and perceptions of participants toward the COVID-19 pandemic. Finally, all researchers worked together to further ensure the trustworthiness of the identified themes and sub-themes. In the following, all themes and sub-themes were analyzed and discussed in detail.

## Results

Five themes and 12 sub-themes concerning experience and perceptions of Chinese university students regarding the COVID-19 pandemic were identified, which were shown in Table 2.

**Table 2:** Results: five themes and 12 their sub-themes.

Themes	Sub-themes
Theme 1 Emotional reactions toward the COVID-19 pandemic	Fear and anxiety at the initial outbreak of the COVID-19 pandemic
	Being relaxed after the effective control of the COVID-19 pandemic
Theme 2 The influence of the COVID-19 pandemic on the participant's learning	Negative aspects of online learning
	Positive aspects of online learning
Theme 3 The influence of the COVID-19 pandemic on the participant's daily life	Family life
	Social life
Theme 4 Participants' positive responses to the COVID-19 pandemic	Prevention and self-care strategies
	Sharing anti-epidemic knowledge with their parents or relatives. Taking part in community prevention and control of the COVID-19 pandemic
Theme 5 China's responses to the COVID-19 pandemic	The Chinese government's moves toward the COVID-19 pandemic
	Medical professional's responses to the COVID-19 pandemic
	Social organization's responses to the COVID-19 pandemic

### Participants' Emotional Reactions Toward the COVID-19 Pandemic

This theme addressed participants' emotional reactions toward the COVID-19 pandemic. Through the detailed analysis of the interview data, it was found that participants' emotional reactions toward the COVID-19 pandemic could be classified into two sub-themes: (1) fear and anxiety in the initial phase of the COVID-19 pandemic; (2) being relaxed after the effective control of the COVID-19 pandemic.

#### Fear and Anxiety at the Beginning of the COVID-19 Pandemic

After the outbreak of the COVID-19 pandemic, university students also experienced extreme fear and worry about their own health and the health of their loved ones. The highly contagious strain of the coronavirus was the main reason why they felt scared.

*In the initial phase of the COVID-19 pandemic, the COVID-19 pandemic spread quickly and infected a large number of people. The number of confirmed coronavirus cases increased exponentially each day. To the worst, a lot of infected people died. I felt very anxious and scared*. Participant 13

*At the beginning of the outbreak, I was very scared because I did not know to what extent the epidemic would develop. It is the first time for me to go through such a serous epidemic*. Participant 8

#### Being Relaxed After the Effective Control of the COVID-19 Pandemic

After the outbreak of the COVID-19 pandemic, central and local governments took a range of strict measures to stop the spread of the COVID-19 pandemic. Meanwhile, strict prevention and control measures were carried out by communities where participants lived. Correspondingly, confirmed cases of the COVID-19 pandemic declined significantly and the COVID-19 pandemic was under control, which make university students feel relaxed.

*Immediately after the outbreak of the COVID-19 pandemic, the Chinese government took a lot of effective measures against the epidemic. Also, the communities where I live did a lot of work to prevent the spread of the epidemic. Finally, the epidemic was well controlled. Now, I feel relaxed*. Participant 4

*The government made a rapid response to the COVID-19 pandemic and the number of confirmed cases gradually reduced, which made me feel relaxed*. Participant 7

### The Influence of the COVID-19 Pandemic on University Students' Learning

China lockdowned all levels of school, which caused interruption in students' classroom learning. Learning moved online, which had significant effects on students' learning ways and efficiency. This theme explored the participants' views on the influence of the COVID-19 pandemic on their learning, which could be further divided into two sub-themes, that is, negative aspects of online learning and positive aspects of online learning.

#### Negative Aspects of Online Learning

To avoid the cross-infection among university students, all universities were closed off and teaching moved online. In this sense, university students had to learn through online platforms. However, online learning made them inaccessible to full communication with their teachers and classmates.

*I'm not comfortable with online classes. I have to see the computer screen every day, and I could not be able to discuss issues with my teachers and classmates fully. I could not adapt myself to online learning*. Participant-8

Due to the lack of face-to-face interaction, students were easily distracted in the course of online learning. Inattentive behavior in online learning could disrupt the learning process and thus negatively influence their learning efficiency.

*I tend to get distracted during the online class and could only memorize a few words in three or four hours*. Participant 2

#### Positive Aspects of Online Learning

Several participants reported that home-based learning could improve their learning efficiency. Some students might be struggling with anxiety in the classroom, which made them have difficulty concentrating in class. In contrast, online learning could downgrade the student's negative emotions and improve their learning efficiency.

*Teachers will ask some students at random to answer questions in classroom teaching, which makes me feel nervous and thus slow down my learning efficiency. In contrast, teachers seldom ask students to answer questions during online learning, which makes me feel relaxed. Thus, I could stay focused while studying*. Participant 5

Online learning could make students learn faster than face-to-face classroom learning. Students can learn at their own pace, going back and re-reading, skipping, or accelerating through concepts as they choose.

*The teacher sent us the recorded teaching video in advance, which allowed me to set my own learning pace. Some of the knowledge has already been learned before, which I can skip. I could thus pay much more attention to the knowledge that I have never known before. I could learn faster online than I would do that in classroom*. Participant 6

### The Influence of the COVID-19 Pandemic on the Participants' Daily Lives

The COVID-19 pandemic kept students stay at home, which has an obvious influence on their daily lives. This theme described how the COVID-19 pandemic influences participant's day-to-day lifestyle. It focused mainly on the influence of the COVID-19 pandemic on the student's family life and social life.

#### Family Life

University students usually lived in campus, which was far away from their own home. Thus, they did not have sufficient time to communicate with their parents. The COVID-19 pandemic left universities in lockdown and students must stay at home, which would keep them in close touch with their parents. Subsequently, their relationships with their parents were enhanced.

*I have more time to communicate with my parents. After dinner, we play games together at home. I feel that the relationship between parents and me is much closer than before*. Participant 13

*During the pandemic, I have more time to communicate with my father. I get a more harmonious relationship with him than before. As a consequence, I have found some strengths of my father that I did not notice before*. Participant 9

The COVID-19 pandemic occurred during the Chinese spring festival, which holds the most important position among all Chinese festivals. During the spring festival, Chinese university students usually spend much time visiting their friends and relatives. The outbreak of COVID-19 isolated university students from their relatives or friends. They had to celebrate the spring festival at their own home.

*My good friends and I studied at different universities in different cities. During Chinese new spring festival, we usually go back to our own hometown. It is a unique opportunity for us to meet each other. However, we cannot meet each other due to the outbreak of the COVID-19 pandemic*. Participant 7

*Due to the outbreak of the epidemic, relative gatherings which are a conventional activity of Chinese new year have been canceled*. Participant 2

Moreover, the COVID-19 pandemic brought about a negative impact on participant's quality of life. At the beginning of the COVID-19 pandemic, participants were not allowed to go outside. They could only leave their houses for going shopping or other urgent needs.

*Because of the restrictions on the frequency and time of outings, shopping was not very convenient. In addition, the epidemic disrupted food supplies. As a consequence, some food we would like to get could not be bought*. Participant 15

*During the epidemic, I usually went shopping one time a week. I could not be able to eat fresh vegetables every day, which led to a decline in the quality of life*. Participant 4

#### Social Life

After the outbreak of the COVID-19 pandemic, China closed off all types of public transportation to minimize the spread of the pandemic. People were only able to drive private vehicles to go shopping or address other urgent things. The unavailability of public transport had a negative influence on students' social life.

*During the COVID-19 pandemic, all types of public transport were closed off, which was very inconvenient for our shopping. We could only drive private vehicles to go shopping*.

On the other hand, the COVID-19 pandemic disturbed students' social contact. Students had planned to take a trip or take part in various types of social activities during the spring festival. The outbreak of the COVID-19 pandemic disrupted their original schedules.

*I originally planned to travel after the spring festival. However, the epidemic disrupted all the plans as scheduled, and I could only stay at home*. Participant 1

### Participants' Responses to the COVID-19 Pandemic

Participants reported that they took a range of measures to fight against the COVID-19 pandemic. This theme demonstrated actions that participants took to combat against the COVID-19 pandemic.

#### Prevention and Self-Care Strategies

Students took a range of self-care strategies to prevent the COVID-19 pandemic and took good care of themselves. Self-care strategies are good for students' mental and physical health and can help them take charge of their lives.

*I follow community's instruction strictly. I try not to go out. I must wear a mask when I have to go outside*. Participant 5

*When I feel uncomfortable, I will have my body checked immediately and establish whether I was affected by the COVID-19 pandemic. This would greatly reduce the possibility that the epidemic will spread to my family*. Participant 12

#### Sharing Anti-epidemic Knowledge With Their Parents or Relatives

University students are open-minded and have a high degree of social responsibility. In this sense, they are very likely to take effective actions toward public emergencies. Almost all participants in this study said that they shared anti-epidemic knowledge with their family members.

*At the very beginning of the COVID-19 pandemic, my parents would not like to wear face mask when they go outside. By constantly telling them the benefits of wear face masks in public, they were used to wearing face masks when they went out to grocery stores or pharmacies*. Participant 7

*I tell my parents and grandparents about the number of new confirmed cases everyday. I also tell them how to avoid being infected by the virus, such washing hands frequently, wearing face masks, never eating wild animals*. Participant 14

#### Taking Part in Community Prevention and Control of the COVID-19 Pandemic

After the outbreak of the COVID-19 pandemic, participants actively participated in the pandemic prevention and control in their own communities.

*As a volunteer, I worked with community staff to prevent the spread of the COVID-19 pandemic in my own community. I handed out brochure to the community residents whereby the preventive measures against the COVID-19 pandemic could be accessible to the community residents*. Participant 6

### China's Responses to The COVID-19 Pandemic

After the outbreak of the COVID-19 pandemic, China took a range of positive actions to deal with the COVID-19 pandemic. This theme illustrated how China responded to the COVID-19 pandemic based on the knowledge of the participants toward what China had done to deal with the COVID-19 pandemic.

#### The Chinese Government's Actions Toward the COVID-19 Pandemic

To stop the spread of the COVID-19 pandemic, all types of public transportation stopped and residents were required to stay at home.

*In my city, all shops were closed off except those selling basic necessities of life or medicine. All public transportation stopped and only a small number of private vehicles were allowed to drive on roads with special permission*. Participant 7

*All types of public transportation were suspended, and roads were blocked. Community officials told us not to go outside without permission*. Participant 11

Due to the severe shortage of hospital beds, a large number of confirmed patients could not be admitted to hospitals. To make all confirmed patients get quick access to medical treatment, Chinese government immediately established “Fangcang” hospitals, which were public facilities such as stadiums and conference centers that had been repurposed for medical care. The Fangcang hospitals played a significant role in the successful control of the COVID-19 pandemic in China.

*Immediately after the outbreak of the COVID-19 pandemic in Wuhan, the Chinese government established a good number of Fangcang hospitals in Wuhan, which could ensure that all confirmed patients could be timely treated. A large number of volunteer medical professionals were assigned to work in Fangcang hospitals*. Participant 8

In terms of the treatment fees of confirmed patients, they were fully covered by China government, which could reduce patents' economic burdens and thus enhance their confidence in fighting against the COVID-19 pandemic.

*As far as I know, hospitals admit the infected patients without charging any fees. The Chinese government fully pays for treatment fees of the infected patients. The Chinese government goes hand in hand with Chinese people to fight against the virus. I am proud of being Chinese*. Participant 5

#### Medical Professionals' Responses to the COVID-19 Pandemic

At the very beginning of the COVID-19 pandemic, there was a severe shortage of medical professionals in Wuhan's hospitals due to the exponential increase of the confirmed patients. As a result, a large number of Chinese medical staff rushed to Wuhan to fight against the COVID-19 pandemic voluntarily.

*Immediately after the outbreak of the COVID-19 pandemic, a large number of volunteer medical staff rushed to Wuhan. Some of them were infected with the COVID-19 pandemic in the process of treating or nursing the confirmed patients. The COVID-19 pandemic in China cannot be well controlled without the selfless devotion of Chinese medical staff*. Participant 12

*After the outbreak of the COVID-19 pandemic, Chinese medical workers actively participate in the fight against the COVID-19 pandemic. Due to the severe shortage of nurses in Wuhan's hospitals, a large number of volunteer nurses headed to Wuhan. I noticed that a number of them volunteered to shave their heads bald before their departure. I am very proud of them and adore them very much*. Participant 16

#### Social Organization's Response to the COVID-19 Pandemic

As the frontline of the COVID-19 pandemic, Wuhan's hospitals ran severely short of medical supplies due to the exponential increase of the confirmed patients. To relieve this difficulty, a number of social organizations provided medical supplies and financial support to the frontline hospitals in Wuhan.

*Hanhong foundation donated a large number of surgical face mask, medical gloves and protective suits to medical professionals who worked in the frontline hospitals in Wuhan. The frontline hospitals in Wuhan also received a good number of medical equipments donated by Hanhong foundation, such as ventilators, ECG monitors and ultrasonic units*. Participant 11

## Discussion

In this section, we will discuss the influence of the pandemic on participant's psychological distress, learning, and daily lives. Also, participant's knowledge of China's actions toward the pandemic will be discussed. The COVID-19 pandemic exerted a significant effect on psychological distress among Chinese university students. At the beginning of the COVID-19 pandemic, all participants showed negative emotions such as fear, anxiety and worry. Some participants even showed extreme fear for the epidemic because they were worried that they would be infected. With the effective control of the pandemic, participants' emotions changed from fear and anxiety to relaxation and confidence. Similar findings were reported by scholars in developed countries, such as America, France and Italy (28–30). It has also been found that university students seem to be more likely to be influenced by mental illnesses during COVID-19 pandemic in France. Specifically, they are susceptible to anxiety, depression and posttraumatic stress (11). Also, there was a significant increase of psychological distress among the university students during COVID-19 pandemic in Italy (28).

Immediately after the outbreak of the COVID-19 pandemic, China lockdowned all its universities and universities teachers were required to teach online through a range of softwares such as Yuketang, Tencent Meeting and Wisdom Tree (31). Online learning could make students learn faster than face-to-face classroom learning. Students could learn at their own pace, going back and re-reading, skipping, or accelerating through concepts as they choose. However, it cannot be denied that online teaching exerted negative influences on university student's learning. For instance, students could not make sufficient discussion with their teachers and classmates in the online classroom. Some students reported that they were easily distracted in the course of online learning. According to our detailed analysis of the interview data, it was identified that there were both advantages and disadvantages of online teaching for university students' online learning. In this study, similar to previous studies, it was found that online learning has its advantages and disadvantages (32).

In addition, the COVID-19 pandemic kept students staying at home, which had a significant influence on their family life. In this sense, students would have more time to stay with their parents, which could harmonize home life and strengthen family relationships. In turn, they would be isolated from their relatives and friends. Measures that have been conducted in many countries to prevent and control the spread of the COVID-19 had a negative impact on interpersonal relationships in general and relative or friend relationship specifically (30). In addition, the COVID-19 pandemic had a severe influence on the university students' social lives. Immediately after the outbreak of the pandemic, China closed off all types of public transportation to minimize the spread of the pandemic, which influenced people's outings negatively. Also, it has been reported that the lives of university students are more likely to be influenced by the COVID-19 among the general population (11).

In response to the COVID-19 pandemic, the Chinese government has taken a range of measures, including extreme lockdowns, establishing Fangcang hospitals and providing national free treatment to the confirmed patients. The COVID-19 pandemic is now well-controlled as a result of China's rapid and effective response to the COVID-19 pandemic. All participants were very satisfied with the way the Chinese government handled the epidemic. This is in line with the results of an existing study, which suggests that most university students were satisfied with the government's actions against COVID-19 pandemic (28).

To the best of our knowledge, this is the first qualitative study to examine how Chinese university students experience and perceive the COVID-19 pandemic. Although much work has been done to strengthen the trustworthiness of this study, there are still several limitations to this study. Due to the limited number of participants, the findings of this study could not be generalized. In addition, only participants who live in Eastern China have been involved in this study. Further research could be expanded to participants who live in other parts of China. Moreover, special attention should be given to poor studuents, who may be more vulnerable during the COVID-19 pandemic.

## Conclusion

The present study demonstrated how the COVID-19 pandemic influences the university students' emotion, learning, and daily lives, as well as how the university students perceive and respond to the pandemic. Although the pandemic had a negative influence on the university students' daily lives and learning, they never surrender to it but taking the initiative to combat against it. Notably, the Chinese government took a range of positive measures to prevent and control the spread of the COVID-19 pandemic. All university students involved in this study were satisfied with the government's actions against the spread of the pandemic. The findings of this study can provide us a better understanding of how the university students perceive the COVID-19 pandemic and to what extent the COVID-19 pandemic has influenced their learning and daily lives.

## Data Availability Statement

The raw data supporting the conclusions of this article will be made available by the authors, without undue reservation.

## Ethics Statement

Ethical review and approval was not required for the study on human participants in accordance with the local legislation and institutional requirements. The patients/participants provided their written informed consent to participate in this study.

## Author Contributions

All authors listed have made a substantial, direct, and intellectual contribution to the work and approved it for publication.

## Funding

This study was supported by Shandong Provincial Philosophy and Social Science Planning Project (2021CYJ08).

## Conflict of Interest

The authors declare that the research was conducted in the absence of any commercial or financial relationships that could be construed as a potential conflict of interest.

## Publisher's Note

All claims expressed in this article are solely those of the authors and do not necessarily represent those of their affiliated organizations, or those of the publisher, the editors and the reviewers. Any product that may be evaluated in this article, or claim that may be made by its manufacturer, is not guaranteed or endorsed by the publisher.
